# Supplementary material for: Emerging Treatments for Disorders of Consciousness in Paediatric Age
Source: Brain Sci. 2022 Jan 31;12(2):198. doi: 10.3390/brainsci12020198 (PMC8870410; doi:10.3390/brainsci12020198)
Supplement: Supplementary file 1 [file brainsci-12-00198-s001.zip › brainsci-1560733-supplementary.pdf]

SUPPLEMENTARY MATERIAL
Commentary: Emerging treatments for disorders of consciousness in paediatric age.

Hassna Irzan[2,1], Marco Pozzi[3], Nino Chikhladze[4], Serghei Cebanu[5], Artashes Tadevosyan[6], Cornelia Calcii[5], Alexander Tsiskaridze[4], Andrew Melbourne[1,2], Sandra Strazzer[3,7], Marc Modat[1] and Erika Molteni[1*].

Affiliations.

1. School of Biomedical Engineering & Imaging Sciences, King's College London, London, United Kingdom.
2. Department of Medical Physics and Biomedical Engineering, University College London, United Kingdom.
3. Scientific Institute IRCCS E. Medea, Acquired Brain Injury Unit, Bosisio Parini, Italy.
4. Faculty of Medicine, Ivane Javakhishvili Tbilisi State University, Georgia.
5. Faculty of Medicine, Nicolae Testemitanu State University of Medicine and Pharmacy, Republic of Moldova.
6. Department of Public Health and Healthcare Organization, Yerevan State Medical University, Armenia.
7. Rehabilitation Service, "Usratuna" Health and Rehabilitation Centre, Juba, South Sudan.

Supplementary Table 1. Summary of previous clinical studies of drug or regenerative treatments for DoC.

Drug	Population	Study type	Sample size	First author	Year	Ref
Amantadine	3-18	Retrospective	118	Green	2004	10.1097/01.phm.0000143400.15346.c8 [1]
Amantadine	6-18	RDBCT, XO	5	McMahon	2009	10.1097/PHM.0b013e3181a5ade3 [2]
Amantadine	5-18	RDBCT, XO	5	Vargus-Adams	2010	10.1016/j.pmrj.2009.10.010 [3]
Amantadine	16	Single case	1	Zao	2020	10.20344/amp.11257 [4]
Amantadine, pramipexole	12-21	RDBCT, PA	10	Patrick	2006	10.1177/08830738060210100901 [5]
Dopaminergic drugs	8-19	Retrospective	10	Patrick	2003	10.1080/0269905031000070279 [6]
Methylphenidate	3-16	Retrospective	10	Hornyak	1997	10.3109/17518429709060937 [7]
Modafinil	15	Single case	1	Dhamapurkar	2017	10.1080/09638288.2016.1236414 [8]

Zolpidem	4-17	RDBCT, XO	3	Snyman	2010	10.1055/s-0030-1269893 [9]
Zolpidem	16	Single case	1	Appu	2014	10.1016/j.pediatrneurol.2013.11.001 [10]
Lorazepam, methylprednisolone	5	Single case	1	Bobele	1999	10.1016/s1071-9091(99)80008-4 [11]
Nerve growth factor	4	Single case	1	Chiaretti	2017	10.1080/02699052.2017.1376760 [12]
Nerve growth factor	7	Single case	1	Chiaretti	2020	10.1007/s00381-020-04590-x [13]
Autologous bone marrow derived mononuclear cells transplantation	2-6	Prospective	5	Liem	2020	10.3389/fped.2020.00564 [14]
Cord blood cells transplantation	1	Single case	1	Jozwiak	2010	10.3727/215517910X536618 [15]
Cord blood cells transplantation	3	Single case	1	Jensen	2013	10.1155/2013/951827 [16]
Amantadine	Adults	Single case	1	Schnakers	2008	10.1136/jnnp.2007.124099 [43]
Amantadine	Adults	RDBCT, PA	184	Giacino	2012	10.1056/NEJMoa1102609 [17]
Amantadine	Adults	Single case	1	Estraneo	2015	10.1007/s00415-015-7771-y [18]
Amantadine	Adults	Prospective	7	Gao	2020	10.1080/02699052.2020.1780315 [19]
Amantadine	Adults	?	142	Kondratieva	2020	10.17116/jnevro2020120121102 [44]
Amantadine + Cerebrolysin	Adults	Retrospective	84	Lee	2020	10.2340/16501977-2654 [20]
Amantadine + TMS	Adults	Prospective	4	Bender Pape	2020	10.1097/HTR.0000000000000634 [21]
Bromocriptinie	Adults	Retrospective	5	Passler	2001	10.1053/apmr.2001.20831 [22]
Levodopa	Adults	Retrospective	5	Matsuda	2005	10.1080/09602010443000588 [23]
Levodopa	Adults	Prospective	7	Ugoya	2010	10.1097/WNF.0b013e3182011070 [24]
Apomorphine	Adults	Single case	1	Fridman	2009	10.1080/02699050802649662 [25]
Apomorphine	Adults	Prospective	8	Fridman	2010	10.3109/02699051003610433 [26]
Selegiline	Adults	Prospective	6	Masotta	2018	10.1017/cjn.2018.315 [27]
Methylphenidate	Adults	Retrospective	22	Martin	2007	10.1097/PHM.0b013e3181154a84 [28]
Psychostimulants	Adults	Retrospective	115	Herrold	2014	10.1155/2014/964578 [29]
Psychostimulants	Adults	Retrospective	48	Barra	2020	10.1177/0885066619841603 [30]

Desipramine, protriptyline	Adults	Retrospective	8	Wroblewski	1993	10.3109/02699059309034962 [31]
Amitriptyline	Adults	Retrospective	3	Reinhard	1996	10.1016/s0003-9993(96)90225-7 [32]
Zolpidem	Adults	Prospective	15	Whyte	2009	10.1097/PHM.0b013e3181a0e3a0 [33]
Zolpidem	Adults	Prospective	16	Machado	2014	10.2174/13816128113196660646 [34]
Zolpidem	Adults	RDBCT, XO, single administration	84	Whyte	2014	10.1097/PHM.0000000000000069 [19]
Zolpidem	Adults	Single case	1	Calabrò	2015	10.1111/pcn.12215 [35]
Zolpidem	Adults	Single case	1	Delargy	2019	10.1080/02699052.2018.1537008 [36]
Zolpidem	Adults	Single case	1	Sayadnasiri	2019	10.1097/WNF.0000000000000362 [37]
Zolpidem, Lorazepam	Adults	Retrospective	146	Zhang	2021	10.3390/brainsci11060726 [38]
Midazolam	Adults	Single case	1	Carboncini	2011	10.3233/RNN-140426 [39]
Midazolam	Adults	Single case	1	Carboncini	2014	10.3233/RNN-140426 [40]
Baclofen	Adults	Prospective	8	Margetis	2014	10.1111/ner.12147 [41]
Baclofen	Adults	Retrospective	5	Sarà	2009	10.1016/j.apmr.2009.01.012 [42]
Ziconotide	Adults	Single case	1	Lanzillo	2016	EJPRM 2016;52(2):263-6 [45]
Modafinil	Adults	Retrospective	24	Dhamapurkar	2017	10.1080/09638288.2016.1236414 [8]
Modafinil	Adults	Single case	1	Formica	2017	10.5664/jcsm.6854 [43]
Sertraline	Adults	Retrospective	35	Danner	2020	https://doi.org/10.37191/Mapsci-2582-4333-2(3)-037 [42]

Bibliography:

1. Green, L.B.; Hornyak, J.E.; Hurvitz, E.A. Amantadine in Pediatric Patients with Traumatic Brain Injury: A Retrospective, Case-Controlled Study. *American Journal of Physical Medicine and Rehabilitation* **2004**, *83*, 893–897, doi:10.1097/01.PHM.0000143400.15346.C8.

2. McMahon, M.A.; Vargus-Adams, J.N.; Michaud, L.J.; Bean, J. Effects of Amantadine in Children with Impaired Consciousness Caused by Acquired Brain Injury: A Pilot Study. *American Journal of Physical Medicine and Rehabilitation* **2009**, *88*, 525–532, doi:10.1097/PHM.0b013e3181a5ade3.

3. Vargus-Adams, J.N.; McMahon, M.A.; Michaud, L.J.; Bean, J.; Vinks, A.A. Pharmacokinetics of Amantadine in Children With Impaired Consciousness Due to Acquired Brain Injury: Preliminary Findings Using a Sparse-Sampling Technique. *PM and R* **2010**, *2*, 37–42, doi:10.1016/j.pmrj.2009.10.010.

4. Almeida, A.F.; Beça, G.; Nunes, R.; Ana, Z.Ã.O. From Vegetative State to Participation: Amantadine As a Trigger of the Rehabilitation Program. *Acta Medica Portuguesa* **2020**, *33*, 604–609, doi:10.20344/amp.11257.

5. Patrick, P.D.; Blackman, J.A.; Mabry, J.L.; Buck, M.L.; Gurka, M.J.; Conaway, M.R. Dopamine Agonist Therapy in Low-Response Children Following Traumatic Brain Injury. *Journal of Child Neurology* **2006**, *21*, 879–885, doi:10.1177/08830738060210100901.

6. Patrick, P.D.; Buck, M.L.; Conaway, M.R.; Blackman, J.A. The Use of Dopamine Enhancing Medications with Children in Low Response States Following Brain Injury. *Brain Injury* **2003**, *17*, 497–506, doi:10.1080/0269905031000070279.

7. Hornyak, J.E.; Nelson, V.S.; Hurvitz, E.A. The Use of Methylphenidate in Paediatric Traumatic Brain Injury. *Developmental Neurorehabilitation* **1997**, *1*, 15–17, doi:10.3109/17518429709060937.

8. Dhamapurkar, S.K.; Wilson, B.A.; Rose, A.; Watson, P.; Shiel, A. Does Modafinil Improve the Level of Consciousness for People with a Prolonged Disorder of Consciousness? A Retrospective Pilot Study. *Disability and Rehabilitation* **2017**, *39*, 2633–2639, doi:10.1080/09638288.2016.1236414.

9. Snyman, N.; Egan, J.R.; London, K.; Howman-Giles, R.; Gill, D.; Gillis, J.; Scheinberg, A. Zolpidem for Persistent Vegetative State - A Placebo-Controlled Trial in Pediatrics. *Neuropediatrics* **2010**, *41*, 223–227, doi:10.1055/s-0030-1269893.

10. Appu, M.; Noetzel, M. Clinically Significant Response to Zolpidem in Disorders of Consciousness Secondary to Anti-N-Methyl-d-Aspartate Receptor Encephalitis in a Teenager: A Case Report. *Pediatric Neurology* **2014**, *50*, 262–264, doi:10.1016/j.pediatrneurol.2013.11.001.

11. Bobele, G.B.; Bale, J. Subacute Encephalopathy in a 5-Year-Old Boy. *Seminars in Pediatric Neurology* **1999**, *6*, 168–172, doi:10.1016/S1071-9091(99)80008-4.

12. Chiaretti, A.; Conti, G.; Falsini, B.; Buonsenso, D.; Crasti, M.; Manni, L.; Soligo, M.; Fantacci, C.; Genovese, O.; Calcagni, M.L.; et al. Intranasal Nerve Growth Factor Administration Improves Cerebral Functions in a Child with Severe Traumatic Brain Injury: A Case Report. *Brain Injury* **2017**, *31*, 1538–1547, doi:10.1080/02699052.2017.1376760.

13. Chiaretti, A.; Eftimiadi, G.; Buonsenso, D.; Rendeli, C.; Staccioli, S.; Conti, G. Intranasal Nerve Growth Factor Administration Improves Neurological Outcome after GBS Meningitis. *Child's Nervous System* **2020**, *36*, 2083–2088, doi:10.1007/s00381-020-04590-x.

14. Liem, N.T.; Chinh, V.D.; Phuong, D.T.M.; Van Doan, N.; Forsyth, N.R.; Heke, M.; Thi, P.A.N.; Nguyen, X.H. Outcomes of Bone Marrow-Derived Mononuclear Cell Transplantation for Patients in Persistent Vegetative State After Drowning: Report of Five Cases. *Frontiers in Pediatrics* **2020**, *8*, 1–10, doi:10.3389/fped.2020.00564.

15. Jozwiak, S.; Habich, A.; Kotulska, K.; Sarnowska, A.; Kropiwnicki, T.; Janowski, M.; Jurkiewicz, E.; Lukomska, B.; Kmiec, T.; Walecki, J.; et al. Intracerebroventricular Transplantation of Cord Blood-Derived Neural Progenitors in a Child with Severe Global Brain Ischemic Injury. *Cell Medicine* **2010**, *1*, 71–80, doi:10.3727/215517910x536618.

16. Jensen, A.; Hamelmann, E. First Autologous Cell Therapy of Cerebral Palsy Caused by Hypoxic-Ischemic Brain Damage in a Child after Cardiac Arrest—Individual Treatment with Cord Blood. *Case Reports in Transplantation* **2013**, *2013*, 1–6, doi:10.1155/2013/951827.

17. Giacino, J.T.; Whyte, J.; Bagiella, E.; Kalmar, K.; Childs, N.; Khademi, A.; Eifert, B.; Long, D.; Katz, D.I.; Cho, S.; et al. Placebo-Controlled Trial of Amantadine for Severe Traumatic Brain Injury. *Survey of Anesthesiology* **2013**, *57*, 216–217, doi:10.1097/01.sa.0000433227.82776.07.

18. Estraneo, A.; Pascarella, A.; Moretta, P.; Loreto, V.; Trojano, L. Clinical and Electroencephalographic on–off Effect of Amantadine in Chronic Non-Traumatic Minimally Conscious State. *Journal of Neurology* **2015**, *262*, 1584–1586, doi:10.1007/s00415-015-7771-y.

19. Snyman, N.; Egan, J.R.; London, K.; Howman-Giles, R.; Gill, D.; Gillis, J.; Scheinberg, A.; Whyte, J.; Rajan, R.; Rosenbaum, A.; et al. Zolpidem and Restoration of Consciousness. *American Journal of Physical Medicine and Rehabilitation* **2014**, *41*, 223–227, doi:10.1097/PHM.0000000000000069.

20. Lee, S.; Lee, H.H.; Lee, Y.; Lee, J. Additive Effect of Cerebrolysin and Amantadine on Disorders of Consciousness Secondary to Acquired Brain Injury: A Retrospective Case-Control Study. *Journal of rehabilitation medicine* **2020**, *52*, jrm00025, doi:10.2340/16501977-2654.

21. Bender Pape, T.L.; Herrold, A.A.; Livengood, S.L.; Guernon, A.; Weaver, J.A.; Higgins, J.P.; Rosenow, J.M.; Walsh, E.; Bhaumik, R.; Pacheco, M.; et al. A Pilot Trial Examining the Merits of Combining Amantadine and Repetitive Transcranial Magnetic Stimulation as an Intervention for Persons with Disordered Consciousness after TBI. *Journal of Head Trauma Rehabilitation* **2020**, *35*, 371–387, doi:10.1097/HTR.0000000000000634.

22. Passler, M.A.; Riggs, R. V. Positive Outcomes in Traumatic Brain Injury-Vegetative State: Patients Treated with Bromocriptine. *Archives of Physical Medicine and Rehabilitation* **2001**, *82*, 311–315, doi:10.1053/apmr.2001.20831.

23. Matsuda, W.; Komatsu, Y.; Yanaka, K.; Matsumura, A. Levodopa Treatment for Patients in Persistent Vegetative or Minimally Conscious States. *Neuropsychological Rehabilitation* **2005**, *15*, 414–427, doi:10.1080/09602010443000588.

24. Ugoya, S.O.; Akinyemi, R.O. The Place of L-Dopa/Carbidopa in Persistent Vegetative State. *Clinical Neuropharmacology* **2010**, *33*, 279–284, doi:10.1097/WNF.0b013e3182011070.

25. Fridman, E.A.; Calvar, J.; Bonetto, M.; Gamzu, E.; Krimchansky, B.Z.; Meli, F.; Leiguarda, R.C.; Zafonte, R. Fast Awakening from Minimally Conscious State with Apomorphine. *Brain Injury* **2009**, *23*, 172–177, doi:10.1080/02699050802649662.

26. Fridman, E.A.; Krimchansky, B.Z.; Bonetto, M.; Galperin, T.; Gamzu, E.R.; Leiguarda, R.C.; Zafonte, R. Continuous Subcutaneous Apomorphine for Severe Disorders of Consciousness after Traumatic Brain Injury. *Brain Injury* **2010**, *24*, 636–641, doi:10.3109/02699051003610433.

27. Masotta, O.; Trojano, L.; Loreto, V.; Moretta, P.; Estraneo, A. Selegiline in Patients With Disorder of Consciousness: An Open Pilot Study. *Canadian Journal of Neurological Sciences* **2018**, *45*, 688–691, doi:10.1017/cjn.2018.315.

28. Martin, R.T.; Whyte, J. The Effects of Methylphenidate on Command Following and Yes/No Communication in Persons with Severe Disorders of Consciousness: A Meta-Analysis of n-of-1 Studies. *American Journal of Physical Medicine and Rehabilitation* **2007**, *86*, 613–620, doi:10.1097/PHM.0b013e3181154a84.

29. Herrold, A.A.; Pape, T.L.B.; Guernon, A.; Mallinson, T.; Collins, E.; Jordan, N. Prescribing Multiple Neurostimulants during Rehabilitation for Severe Brain Injury. *Scientific World Journal* **2014**, *2014*, doi:10.1155/2014/964578.

30. Barra, M.E.; Izzy, S.; Sarro-Schwartz, A.; Hirschberg, R.E.; Mazwi, N.; Edlow, B.L. Stimulant Therapy in Acute Traumatic Brain Injury: Prescribing Patterns and Adverse Event Rates at 2 Level 1 Trauma Centers. *Journal of Intensive Care Medicine* **2020**, *35*, 1196–1202, doi:10.1177/0885066619841603.

31. Wroblewski, B.; Glenn, M.B.; Cornblatt, R.; Joseph, A.B.; Suduikis, S. Protriptyline as an Alternative Stimulant Medication in Patients with Brain Injury: A Series of Case Reports. *Brain Injury* **1993**, *7*, 353–362, doi:10.3109/02699059309034962.

32. Reinhard, D.L.; Whyte, J.; Sandel, M.E. Improved Arousal and Initiation Following Tricyclic Antidepressant Use in Severe Brain Injury. *Archives of Physical Medicine and Rehabilitation* **1996**, *77*, 80–83, doi:10.1016/S0003-9993(96)90225-7.

33. Whyte, J.; Myers, R. Incidence of Clinically Significant Responses to Zolpidem among Patients with Disorders of Consciousness: A Preliminary Placebo Controlled Trial. *American Journal of Physical Medicine and Rehabilitation* **2009**, *88*, 410–418, doi:10.1097/PHM.0b013e3181a0e3a0.

34. Machado, C.; Estévez, M.; Rodríguez, R.; Pérez-Nellar, J.; Chinchilla, M.; DeFina, P.; Leisman, G.; Carrick, F.R.; Melillo, R.; Schiavi, A.; et al. Zolpidem Arousing Effect in Persistent Vegetative State Patients: Autonomic, EEG and Behavioral Assessment. *Current pharmaceutical design* **2014**, *20*, 4185-4202.

35. Wysokiński, A.; Kolińska, J. Rapidly Developing and Self-Limiting Eosinophilia Associated with Clozapine. *Psychiatry and Clinical Neurosciences* **2015**, *69*, 122, doi:10.1111/pcn.12208.

36. Delargy, M.; O'Connor, R.; McCann, A.; Galligan, I.; Cronin, H.; Gray, D.; O'Toole, C. An Analysis of the Effects of Using Zolpidem and an Innovative Multimodal Interdisciplinary Team Approach in Prolonged Disorders of Consciousness (PDOC). *Brain Injury* **2019**, *33*, 242–248, doi:10.1080/02699052.2018.1537008.

37. Sayadnasiri, M.; Rezvani, F. Treatment of Catatonia in Frontotemporal Dementia: A Lesson from Zolpidem Test. *Clinical Neuropharmacology* **2019**, *42*, 186–187, doi:10.1097/WNF.0000000000000362.

38. Zhang, B.; O'brien, K.; Won, W.; Li, S. A Retrospective Analysis on Clinical Practice-Based Approaches Using Zolpidem and Lorazepam in Disorders of Consciousness. *Brain Sciences* **2021**, *11*, doi:10.3390/brainsci11060726.

39. Carboncini, M.C.; Piarulli, A.; Virgillito, A.; Arrighi, P.; Andre, P.; Tomaiuolo, F.; Frisoli, A.; Bergamasco, M.; Rossi, B.; Bonfiglio, L. A Case of Post-Traumatic Minimally Conscious State Reversed by Midazolam: Clinical Aspects and Neurophysiological Correlates. *Restorative Neurology and Neuroscience* **2014**, *32*, doi:10.3233/RNN-140426.

40. Carboncini, M.C.; Piarulli, A.; Virgillito, A.; Arrighi, P.; Andre, P.; Tomaiuolo, F.; Frisoli, A.; Bergamasco, M.; Rossi, B.; Bonfiglio, L. A Case of Post-Traumatic Minimally Conscious State Reversed by Midazolam: Clinical Aspects and Neurophysiological Correlates. *Restorative Neurology and Neuroscience* **2014**, *32*, 767–787, doi:10.3233/RNN-140426.

41. Margetis, K.; Korfias, S.I.; Gatzonis, S.; Boutos, N.; Stranjalis, G.; Boviatsis, E.; Sakas, D.E. Intrathecal Baclofen Associated with Improvement of Consciousness Disorders in Spasticity Patients. *Neuromodulation* **2014**, *17*, 699–704, doi:10.1111/ner.12147.

42. Sarà, M.; Pistoia, F.; Mura, E.; Onorati, P.; Govoni, S. Intrathecal Baclofen in Patients With Persistent Vegetative State: 2 Hypotheses. *Archives of Physical Medicine and Rehabilitation* **2009**, *90*, 1245–1249, doi:10.1016/j.apmr.2009.01.012.

43. Formica, F.; Pozzi, M.; Avantaggiato, P.; Molteni, E.; Arrigoni, F.; Giordano, F.; Clementi, E.; Strazzer, S. Disordered Consciousness or Disordered Wakefulness? The Importance of Prolonged Polysomnography for the Diagnosis, Drug Therapy, and Rehabilitation of an Unresponsive Patient with Brain Injury. *Journal of Clinical Sleep Medicine* **2017**, *13*, 1477–1481, doi:10.5664/jcsm.6854.
